# Staged revision still works for chronic and deep infection of total elbow arthroplasty?

**DOI:** 10.1051/sicotj/2022019

**Published:** 2022-05-26

**Authors:** Jae-Man Kwak, Sang-Pil So, In-Ho Jeon

**Affiliations:** 1 Department of Orthopedic Surgery, Uijeongbu Eulji Medical Center, College of Medicine, Eulji University Uijeongbu 11759 South Korea; 2 Department of Orthopedic Surgery, Asan Medical Center, College of Medicine, Ulsan University Seoul 05505 South Korea

**Keywords:** Staged surgery, Infection, Total elbow arthroplasty, Revision, Total elbow replacement

## Abstract

*Purpose*: Infected total elbow arthroplasty (TEA) is challenging. We evaluate the clinical and radiologic outcomes for chronic and deep infection of TEA with two-stage revision surgery. *Methods*: A total of 10 elbows were included in the study. The mean age was 69.1 ± 15 years (range, 34–83 years). The mean follow-up was 62 (range, 24–108) months. The clinical outcomes were assessed using a visual analog scale (VAS), range of motion (ROM) arc, and Mayo elbow performance score (MEPS). Moreover, radiographic outcomes, time to revision, pathogenic bacteria, preoperative complications, and disease period were evaluated. *Results:* Mean preoperative VAS score of 6.1 had improved to 3.3. Mean preoperative ROM was 68° (flexion-extension), which improved to 86.7°. Mean preoperative MEPS was 46 (range, 0–70), which improved to 75.5 (range, 35–85). The mean disease duration was 8.4 months (range, 5–20 months). The most common causative organism was *methicillin-resistant Staphylococcus aureus*. The second revision rate was 80% at the final follow-up. Radiographic outcome at final follow-up showed that 3 (30%) of 10 patients exhibited radiolucency evidence around the components. Three patients showed nonprogressive radiolucency around the implant interfaces without other indications of infection at the most recent follow-up. *Conclusion*: In patients with chronic and deep infection of TEA, two-stage revision can be an affordable option for eradication of the infection, relieving pain, and restoring joint function. However, the high second revision rate owing to bone and soft-tissue deficits remains a critical issue.

**Level of evidence:** Level IV, Case series, Treatment study

## Introduction

Total elbow arthroplasty (TEA), which has been refined and popularized during the last four decades [1], is effective for treating patients with a severe arthritic elbow, including rheumatoid arthritis, osteoarthritis, and non-salvageable intra-articular elbow fractures [2]. However, TEA is associated with a reported complication rate of up to 62% [3–6], which has led to an increasing number of TEA revisions. When TEA becomes infected, the aim is to eradicate the infection foci by local control (staged surgical interventions involving implant removal and reimplantation) and systemic control (antibiotic treatment). Despite the prevalence of TEA, there is limited literature on optimally managing the infected TEA [7]. Therefore, treatment options for infected TEA have been modeled after protocols for managing periprosthetic hip and knee infections [8–11]. Two-stage revision has been accepted as the standard for treating chronic periprosthetic hip and knee infections and has consequently become a procedure of interest in managing infected TEA [9, 12, 13]. However, mid-term or long-term clinical outcome for two-stage revision has been scarcely reported in previous literature [8, 14]. The purpose of the present study is: (1) to report the mid-term clinical outcome of two-stage revision and (2) to evaluate the radiologic outcome, including periprosthetic radiolucency and significant bone loss that require special management such as allograft prosthetic composite (APC) reconstruction. We hypothesized that the two-stage revision treatment would be affordable in eradicating infection and improving clinical outcomes, but a high complication rate and significant bone loss that could happen during a surgical procedure or due to itself need special attention since it required additional procedure for covering the loss.

## Materials and methods

We obtained Institutional Review Board approval prior to study initiation. We conducted a retrospective review of 12 patients who underwent revision surgery for infected TEA from 2010 to 2017 at tertiary referral centers. We excluded two cases with superficial wound infection that could be managed by antibiotics and wound debridement without implant replacement surgery. A total of 10 elbows were included in the study. The mean age was 69.1 ± 15 years (range, 34–83 years). The mean follow-up was 62 (range, 24–108) months. The inclusion criteria were as follows: (1) documented infection after TEA, (2) chronic infection (>4 weeks) [15], and (3) available medical information for more than 2 years of follow-up. Deep infection was diagnosed according to infection standards after artificial joint implantation, as proposed by the Musculoskeletal Infection Society [16] and based on the diagnostic standard for culture-negative prosthetic joint infections proposed by Osmon et al. [17].

Both clinical and radiographic outcomes were assessed at the final follow-up. The clinical outcomes were evaluated using the visual analog scale (VAS), range of motion (ROM), and the Mayo elbow performance score (MEPS). For the remaining cases of TEA infections, we recorded the causative organism shown in the culture results, the disease period defined by the interval from symptom onset to patient discharge; implant loosening, which was observed intraoperatively; complications after revision surgery; serum inflammatory markers (erythrocyte sedimentation rate or serum C-reactive protein concentration [CRP]); and time to second revision surgery.

The mean interval time from primary to revision surgery to suspected infection was 70.3 ± 46.5 months. The mean time from the suspected infection to revision surgery was 3.1 ± 1.2 months. The mean disease period, which is the interval period from the first visit with the suspected infection to the time of patient discharge, was 8.4 ± 4.3 months (range, 5–20 months). The preoperative and the intraoperative cultures were used to determine the causative organism. The most common infectious organism was methicillin-resistant *Staphylococcus aureus* (MRSA) (4 of 10 cases) (Table 1).

**Table 1:** Summary of the results of clinical and serologic workup.

	Sex	Age	Reason of TEA	Time to infection (mo)	WBC (10^9^/L)	ESR (mm/h)	CRP (mg/L)	Causative organism	Preop VAS	Preop ROM	Preop MEPS
1	F	59	RA	101	12,000	120	4.5	*Pseudomonas aeruginosa*	8	75	45
2	F	65	OA	108	9500	105	8.5	*Staphylococcus epidermidis*	8	45	40
3	F	79	RA	72	18,000	98	9.5	MRSA	7	75	55
4	M	83	OA	16	9900	112	10.2	MRSA	7	50	35
5	F	82	RA	19	19,400	104	9.0	MSSA	5	80	45
6	F	73	OA	33	9500	76	12.4	MSSA	4	70	50
7	M	69	RA	164	13,000	95	4.5	no growth	5	50	45
8	F	78	RA	84	7300	46	5.9	*Staphylococcus haemolyticus*	5	90	55
9	M	34	RA	36	8800	69	3.4	MRSA	6	75	45
10	F	69	RA	70	11,900	92	7.5	MRSA	6	70	45

Mo, months; Time to infection, interval time from index arthroplasty to suspected symptom; Preop, preoperative; RA, rheumatoid arthritis; OA, osteoarthritis; MRSA, methicillin resisted *Staphylococcus aureus*; MSSA, methicillin sensitive *Staphylococcus aureus*.

### Two-stage revision surgery for periprosthetic infection

**Figure 1:**
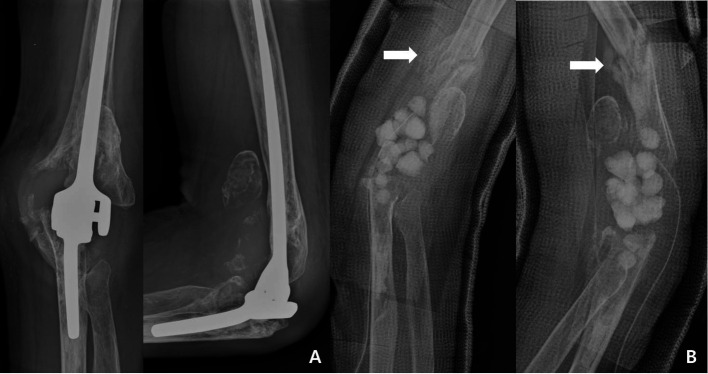
First-stage revision surgery. (A) Chronic and deep infection. Part of the distal humerus and the proximal ulnar was absorbed due to chronic infection with polyethylene wear. (B) Implant removal and insertion of antibiotic cement bead. Fracture occurred during the cement removal.

First-stage surgery included the removal of the infected prosthesis. Meticulous removal of all cement and any suspicious infected tissue, including the synovial membrane, was performed. Cultures were obtained from all implants, and joint fluid and infected tissues were additionally sampled. An antibiotic cement spacer (5 g of gentamicin, 1 g of vancomycin, and 1 g of ceftriaxone per 40 g of cement) was inserted after explantation (Figure 1). We administered an optimum dosage of intravenous antibiotics according to pathogen after an antibiotic susceptibility test. A consultation with an infectious disease specialist was part of the standard protocol. All patients received at least 6 weeks of intravenous antibiotics with radiographic surveillance and inflammatory serology monitoring using erythrocyte sedimentation rate and CRP for evidence of recurrent infection. Once the infection was considered to be clinically and radiographically eradicated, and after the serology results had stabilized, the patients underwent removal of the cement spacer. Additional tissue samples from the medullary canal were obtained for follow-up sensitivity testing. We biopsied a frozen tissue sample to confirm neutrophil and white blood cell counts and prepare for the reimplantation procedure. Any positive result nullified the reimplantation procedure and resulted in the implantation of another antibiotic cement spacer. If successful eradication of infection was confirmed intraoperatively by frozen section and by the absence of obvious infection based on the surgeon’s assessment, reimplantation of the arthroplasty was performed.

### Management of significant bone loss

**Figure 2:**
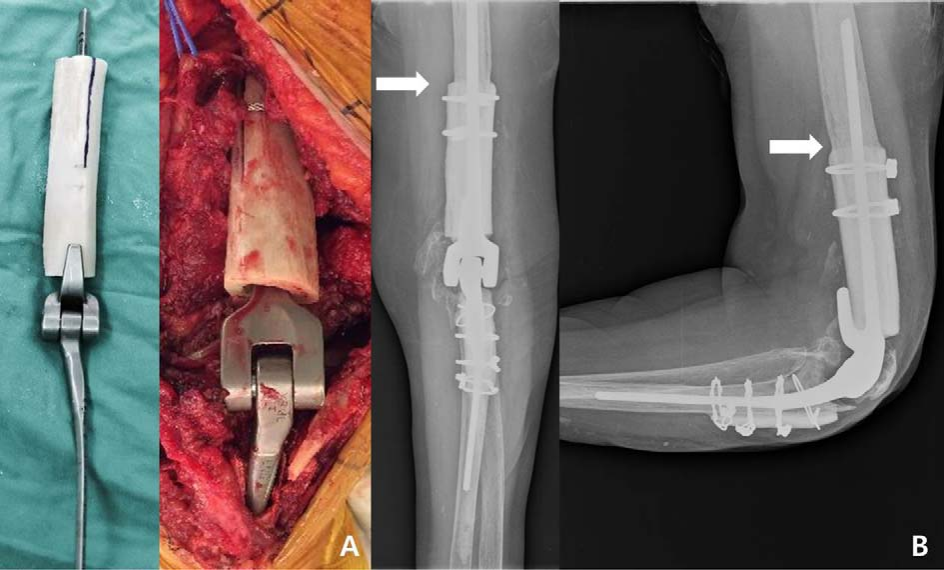
Second-stage revision surgery using APC. (A) Modified type I APC for management of bone defect. (B) Plain radiograph at the time of final follow-up. Bone union was observed between the host and the allo-bone graft.

Morrey et al. [18] reported three specific types of reconstruction techniques using allograft-prosthetic composite (APC) to manage the bone loss. Type I involves the intussusception of APC into the host bone (intussusception type), type II is the insertion of the distal aspect of the stem into the host canal with the strut-like extension of the graft coapted externally to the cortex, and type III is side-to-side contact between the cortices of the APC and the host bone. In the present study, if the host bone stump was too narrow or thin to inset the APC, we modified the APC with a reverse intussusception technique (Figure 2) rather than using a type I technique. During this modified type I technique, after fitting the implant into the allograft bone, some inner medullary canal space was cleared with a burr and longitudinal cleavage to allow placement of the host bone between the stem and the grafted bone. We commonly add wiring to enhance the contact area and fixation between the bones and the implant.

### Statistical analysis

The data were analyzed and are reported as mean ± standard deviation. The preoperative and postoperative clinical outcomes were compared using a paired *t*-test. Statistical significance was set at *P* < 0.05.

## Results

### Clinical outcome

The overall scores for ROM (preoperative to final follow-up: 68 ± 14.7° to 86.7 ± 28.2°, *P* < 0.01), MEPS (46.1 ± 6.1 to 75.5 ± 6.9, *P* < 0.01), and VAS (6.1 ± 1.4 to 3.3 ± 0.9, *P* < 0.01) were improved (Table 2). The main complication rate required for the second revision surgery rate was 80% (8 of 10) at the time of final follow-up, including three cases of aseptic loosening, one case of periprosthetic fracture, and four cases of infection.

**Table 2:** Individual patient outcomes after 2-stage revision for infected TEA.

	Sex	Age	F/U (mo)	Underlying disease	Disease period (mo)	Complication	2nd revision	Time to 2nd revision (mo)	Final VAS	Final ROM	Final MEPS
1	F	59	57	HTN, HBV	20	Aseptic loosening	One staged (APC)	41	3	80	75
2	F	65	39	DM, HTN	9	Aseptic loosening	One staged	18	5	85	65
3	F	79	108	DM, HTN, Osteoporosis	6	Periprosthetic fracture	One staged (APC)	89	2	100	75
4	M	83	74	Cancer, HTN,DM	9	Infection	2-staged (APC)	60	5	85	65
5	F	82	41	Cancer, HBV HTN,DM	7	Infection	2-staged	18	3	85	75
6	F	73	24	HTN, DM, Osteoporosis	9	Infection	2-staged (APC)	9	3	80	75
7	M	69	24	DM, Cancer	6	–	–	–	3	80	85
8	F	78	52	TB,DM	5	–	–	–	3	90	85
9	M	34	96	DM, Osteoposis	5	Aseptic loosening	One staged	24	3	95	80
10	F	69	105	DM, HTN, Osteoposis	8	Infection	One staged (resection)	37	3	–	75

Mo, months; Disease period, interval time from first visit with the symptom to discharge for 2-stage surgery; HTN, hypertension; HBV, hepatitis type B; DM, diabetes mellitus; TB, tuberculosis; APC, allograft-prosthesis composite graft; Resection, resection arthroplasty.

### Radiographic outcome

The radiographic outcome at the time of final follow-up showed that three (30%) of 10 patients exhibited evidence of radiolucency around the components. Three patients showed nonprogressive radiolucency around the implant interfaces without other indications of infection at the most recent follow-up.

## Discussion

Managing chronic and deep infection of TEA remains a challenge, and the consensus on the strategy for infected TEA is still not fully discussed. In our study, two-stage revision surgery achieved satisfactory clinical outcomes at the time of final follow-up, but high complication and revision rate should be addressed.

The limitation of the present study includes that this study is not designed as a comparison study with a limited number of cases (10 cases). However, considering the rarity of the clinical entity [19, 20], this study gives valuable information, including prognosis and strategy of management for deep infection of TEA.

Several findings in our study should be discussed that explain why infected TEA is challenging. First, the complication rate after revision surgery was relatively high. Second, we found high comorbidity rates in patients with TEA infections. Third, MRSA was the most common causative bacteria. The choice of antibiotics is important. Fourth, a proper surgical technique, including allograft-prosthetic composite, should be considered for the management of significant bone loss. All these factors should be considered by surgeons when they decide to perform revision surgery for infected TEA.

First, a high complication rate and a second revision rate are concerns, and they seem to be related to the loss of regeneration of infected bone. Once the bone is infected, *S. aureus* tends to destroy bony structures from the medullary canal by promoting osteoclastogenesis [21, 22]. If the treatment to control or eradicate the infection is delayed, this reaction will be accelerated, induce implant loosening, and increase the chance of breakages secondary to weakened bony structures after revision surgery. In our study, all the patients had a chronic infection, in which the mean interval time from symptom to hospital visit was 3.2 months because the majority of the cases were referral patients from other hospitals. Moreover, incomplete resection of infected bony structures and improper use of antibiotics during the primary management stage can increase the rate of recurrent infections. These issues prolong total hospitalization and lead to worse clinical outcomes, including pain and reduced MEPSs.

Second, it seems that high comorbidity could affect the incidence of infection and complications as well. Several published studies have discussed the relationship between infection and comorbidity rates [6, 23, 24]. The control of general comorbidities and the prevention of infections should be emphasized postoperatively. Among those comorbidities, diabetes – especially when uncontrolled – is a significant risk factor for periprosthetic joint infection. All of our patients also have diabetes, and three patients have uncontrolled diabetes. This finding supports that diabetes is a risk factor for infected TEA. The World Health Organization and the Centers for Disease Control and Prevention recently published guidelines for preventing periprosthetic joint infection [25]. According to the guidelines, postoperative glucose levels should be maintained at <180 mg/dL because even nondiabetic patients who develop hyperglycemia postoperatively have a significantly increased risk [26].

Third, accurate microorganism identification during the initial treatment stage is critical for proper treatment. However, culture-negative rates are non-negligible, making infected TEA more difficult to treat [27]. In our study, there was one culture-negative case of nine cases of TEA infection who received empirical antibiotics before the culture results were analyzed. However, it has been reported that even in the context of a negative culture, using antibiotics can help eradicate infections [28]. As the *Staphylococcus* species are the most common microorganisms implicated in cases of TEA infections, many surgeons choose cephalosporin agents [29]. Unfortunately, cephalosporin is not generally successful because of the high incidence of MRSA, which was the most common organism in our study. Vancomycin has been suggested as an alternative antibiotic in situations where MRSA infections are suspected [30]. In addition, *S. epidermidis* appears to have a more virulent course because of its ability to produce unrelenting biofilms [31]. Morrey and Bryan reported that infection with *S. epidermidis* led to six of seven failures in retained-implant and staged-revision groups [32]. Antibiotic-impregnated cement is commonly used during the index procedure, which may reduce the rate of infection from 11% to 5% [19].

Fourth, the appropriate selection of surgical strategy plays an important role, regardless of the culture findings. Irrigation and debridement with implant retention and one- and two-stage revisions are viable options [30]. Unlike lower limb arthroplasty, antibiotic spacers are not commercially available. Monoblock cement spacers do not allow elbow movements and are associated with severe stiffness and scarring, leading to poor clinical outcomes. Articulating cement spacer has been tried with satisfactory outcomes [33]. IDSA guidelines [17] indicate that for prosthetic joint infections, patients with well-fixed prostheses, without a sinus tract, who are within approximately 30 days of prosthesis implantation or <3 weeks from infectious symptom onset should be considered for debridement with prosthesis retention. Single- or direct-exchange strategies are not common but may be considered in patients with good soft-tissue envelopes, provided that the pathogens are known preoperatively and are susceptible to oral antimicrobials with excellent oral bioavailability. However, it is not simply applied to revision TEA because the elbow lacks a barrier from the skin to the bone that increases the infection risk, especially for trauma, diabetes, and dermatologic disease around the elbow. Considering such a high complication rate of two-stage revision surgery, implant retention only or single-stage surgery is not recommended. Two-stage surgery is more common and is indicated in patients who are not candidates for single-stage reimplantation and are medically fit to undergo multiple surgeries based on the existing soft tissue and bone defects. In our study, all revision surgeries were performed following two-stage reimplantation procedures.

Throughout all the procedures (pre-, intra-, and postoperatively), the use of meticulously aseptic techniques is essential. This includes the careful resection of all suspicious tissue. Information obtained from preoperative magnetic resonance imaging may help determine the ideal resection margin. Whenever surgeons resect suspicious tissue, the margin of resection should be wider than expected, as in tumor surgery. Synthetic implants designed with antibacterial properties are also advantageous for minimizing the risk of infection. Advanced implants take advantage of material technologies like controlled-antibiotic release systems, silver release systems, calcium-based anti-loaded biofilm, and silica-based antibiotic coatings [34].

## Conclusion

In patients with chronic and deep infection of TEA, two-stage revision can be an affordable option for eradication of the infection, relieving pain, and restoring joint function. However, the high second revision rate owing to bone and soft-tissue deficits remains a critical issue.
